# A Multifaceted Giant Protein Microtubule-Actin Cross-Linking Factor 1

**DOI:** 10.3390/ijms26073204

**Published:** 2025-03-30

**Authors:** Chung-Ming Lin, Ru-Huei Fu, Hui-Jye Chen

**Affiliations:** 1 Department of Biotechnology, School of Health and Medical Engineering, Ming Chuan University, Taoyuan 33348, Taiwan; cml@mail.mcu.edu.tw; 2 Translational Medicine Research Center, China Medical University Hospital, Taichung 40447, Taiwan; rhfu@mail.cmu.edu.tw; 3 Ph.D. Program for Aging, China Medical University, Taichung 40402, Taiwan; 4 Graduate Institute of Biomedical Sciences, China Medical University, Taichung 40402, Taiwan

**Keywords:** microtubule-actin cross-linking factor 1, MACF1, ACF7, spectraplakin, cancer, neuronal disease, mental disease, osteoporosis

## Abstract

Microtubule-actin cross-linking factor 1 (MACF1), also known as actin cross-linking family protein 7 (ACF7), is a giant cytolinker protein with multiple conserved domains that can orchestrate cytoskeletal networks of actin and microtubules. MACF1 is involved in various biological processes, including cell polarity, cell–cell connection, cell proliferation, migration, vesicle transport, signal transduction, and neuronal development. In this review, we updated the physiological and pathological roles of MACF1, highlighting the components and signaling pathways involved. Novel evidence showed that MACF1 is involved in diverse human diseases, including multiple neuronal diseases, congenital myasthenic syndrome, premature ovarian insufficiency, spectraplakinopathy, osteoporosis, proliferative diabetic retinopathy, and various types of cancer. We also reviewed the physiological roles of MACF1, including its involvement in adhesome formation, bone formation, neuronal aging, and tooth development. In addition, MACF1 plays other roles, functioning as a biomarker for the prediction of infections in patients with burns and as a marker for genome selection breeding. These studies reinforce the idea that MACF1 is a bona fide versatile, multifaceted giant protein. Identifying additional MACF1 functions would finally help with the treatment of diseases caused by MACF1 defects.

## 1. Introduction

**Figure 1:**
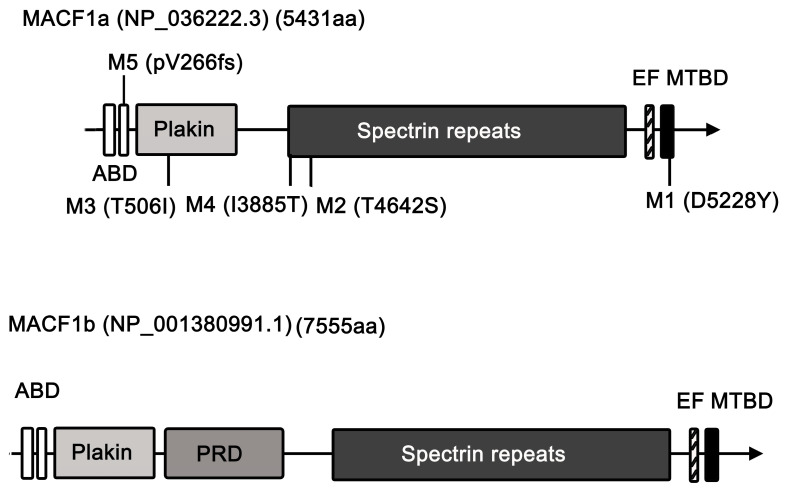
Diagram of human MACF1a and MACF1b isoforms. Domain structures were assigned based on conserved domain analyses on mouse MACF1a and MACF1b. ABD, actin-binding domain, which contains two calponin homology domains. Plakin, plakin domain. PRD, plectin-repeat (plakin-repeat) domain. EF, Ca^2+^-binding EF-hand domain. MTBD, microtubule-binding domain, which contains GAR and GSR domains. Novel mutations mentioned in this review are assigned M1, M2, M3, M4, and M5 on the graph.

MACF1 (microtubule-actin cross-linking factor 1)—also known as actin cross-linking factor 7 (ACF7) [1]—is a member of the spectraplakin protein family [2,3], which comprises the vertebrate members MACF1, BPAG1 (bullous pemphigoid antigen 1), and zebrafish Magellan, and the invertebrate members *Caenorhabditis elegans* VAB-10 and *Drosophila melanogaster* short stop (shot)/Kakapo. Spectraplakin is a giant protein that harbors conserved domains that can bind microfilaments, microtubules, and intermediate filaments, and hence can regulate dynamic cytoskeletal networks. As a spectraplakin member, MACF1 is a conserved protein that consists of multiple domains, which include the N-terminal F-actin-binding calponin homology (CH) domains (ABDs), the plakin domain, the spectrin repeats (SRs), two calcium-binding EF hands (EF), and the C-terminal microtubule-binding domain (MTBD) (Figure 1) [3,4,5]. Alternatively spliced isoforms with additional structures are also discovered in MACF1 [3,4,5,6]. One long isoform, MACF1b, was discovered to possess additional plakin-repeat domains that are located between the plakin domain and the spectrin-repeat domain of MACF1 (now referred to as MACF1a). Research has shown that MACF1b is ubiquitously expressed, can target the Golgi apparatus, and is believed to be involved in the maintenance of the Golgi apparatus structure [7]. Further proteomics analysis of urine samples from patients with cachectic gastro-esophageal cancer revealed that MACF1b could function as a candidate biomarker of cancer cachexia [8].

The multiple-domain nature of MACF1 reveals its diversified functions as it participates in cell polarity, cell–cell connection, cell proliferation, migration, vesicle transport [9,10], dendritic and axonal development [11], and signal transduction such as Wnt signaling [4,12]. MACF1 is also involved in pathological conditions, which include neuromuscular diseases [13], neurodegenerative diseases [13], and cancers [14].

**Figure 2:**
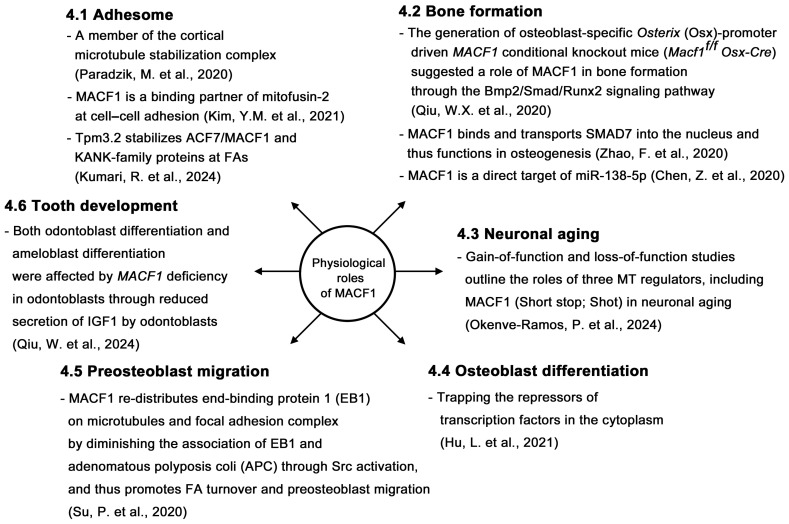
Physiological roles of MACF1. The physiological roles of MACF1 include its participation in adhesome architecture [19,20,21], bone formation [22,23], neuronal aging [24], osteoblast differentiation [25], preosteoblast migration [26], and tooth development [27]. The reference is cited within the parenthesis.

**Figure 3:**
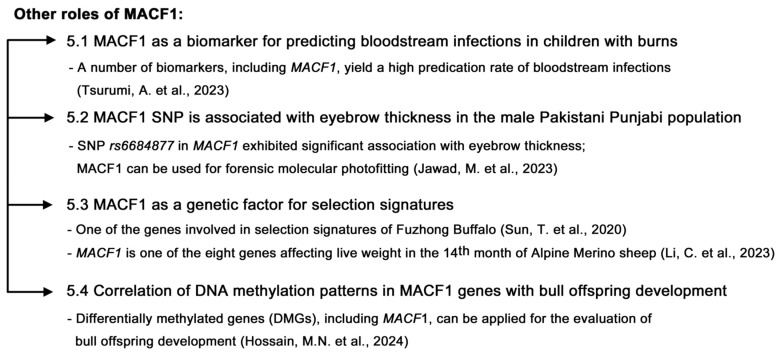
Other roles of MACF1. (1) *MACF1* can function as a biomarker for predicting bloodstream infections in children with burns, and these biomarkers—including *MACF1*—will help with suitable treatments before infection occurs [28]. (2) *MACF1* SNP is associated with eyebrow thickness in the male Pakistani Punjabi population. *MACF1* can be used for forensic molecular photofitting [29]. (3) MACF1 serves as a genetic factor for selection signatures [30,31]. (4) *MACF1* can serve as a differentially methylated gene (DMG) involved in embryonic development after the impact of cold exposure on DNA methylation patterns in cattle sperm. These eight isolated DMGs, including *MACF1*, can be applied for the evaluation of bull offspring development [32].

**Figure 4:**
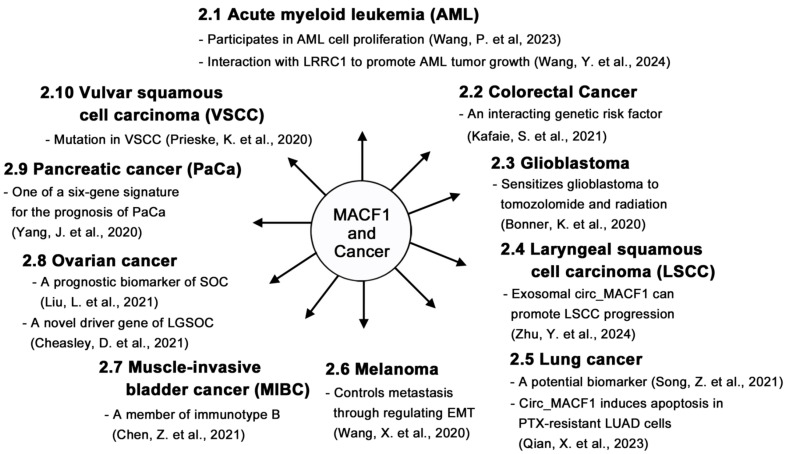
Participation of MACF1 in various cancers. MACF1 is involved in various cancers, including acute myeloid leukemia [33,34], colorectal cancer [35], glioblastoma [36], laryngeal squamous cell carcinoma [37], lung cancer [38,39], melanoma [40], muscle-invasive bladder cancer [41], ovarian cancer [42,43], pancreatic cancer [44], and vulvar squamous cell carcinoma [45]. The possible role of MACF1 in each cancer is highlighted.

**Figure 5:**
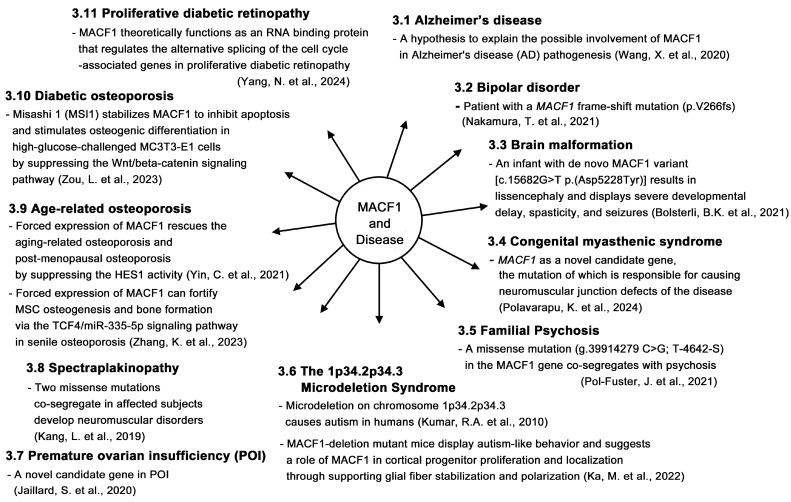
Possible role of MACF1 in various diseases. MACF1 is found to be involved in various diseases, including Alzheimer’s disease [46], bipolar disorder [18], brain malformation [15], congenital myasthenic syndrome [47], familial psychosis [16], microdeletion syndrome [48,49], premature ovarian insufficiency [50], spectraplakinopathy [17], age-related osteoporosis [51,52], diabetic osteoporosis [53], and proliferative diabetic retinopathy [54]. The possible role of MACF1 in individual diseases is highlighted.

In this review, we searched for studies conducted on MACF1 and ACF7 in recent years and reviewed the progress of research on this protein. For the detailed structure and function, biochemistry, and pathological studies, please refer to the seminal review article by Cusseddu et al. [4]. We intended to review the recent discovery of MACF1, focusing on some papers that are not covered by Cusseddu et al. Studies showed that MACF1 participates in multiple physiological processes (Figure 2) and functions as a biomarker (Figure 3). MACF1 is also involved in pathological conditions, including various types of cancer (Figure 4) and other human diseases (Figure 5). Mutations in MACF1 that cause pathological defects are listed in Table 1 and Figure 1. We collected and compiled updated works on MACF1 and hope that this review can initiate more studies on this remarkable gigantic protein.

**Table 1:** MACF1 mutations and related diseases.

Subject No.	Mutation (Variant)	Domain (Region)	Symptoms of Disease	Reference
M1	[c.15682 G>T p. (Asp5228Tyr)]	MTBD	Lissencephaly with brainstem hypoplasia and dysplasia	[15]
M2	(g.39914279 C>G p. T-4642-S) Missense mutation	Spectrin repeats	Familial psychosis	[16]
M3, M4	[c.1517 C>T (p.Thr506Ile)] and [c.11654 T>C (p. Ile3885Thr)] Heterozygous missense mutation	M3, plakin domain M4, spectrin repeats	Spectraplakinopathy type I: progressive spastic tetraplegia, dystonia, joint contracture, feeding difficulty, and developmental delay	[17]
M5	A frame-shift mutation (p.V266fs)	Truncation after ABD	Bipolar disorder	[18]

## 2. Roles of MACF1 in Cancers

### 2.1. Acute Myeloid Leukemia (AML)

Acute myeloid leukemia (AML), a hematologic malignancy that affects older adults—with a median age at diagnosis of 68 years—is associated with a high mortality [55]. Although advancements in diagnosis and new approaches have led to improved outcomes, novel evidence will help with the treatment of the disease. One study revealed the involvement of MACF1 in AML and showed that MACF1 was upregulated in AML patients and AML cells. Poor overall survival rates were observed in patients with high MACF1. MACF1 depletion decreased AML cell proliferation, decreased Runx2 expression, and repressed the activation of PI3K/Akt signaling, suggesting that MACF1 participates in AML cell proliferation through activating the PI3K/Akt signaling pathway and increasing Runx2 expression [33]. Another study revealed that the leucine-rich repeat-containing protein 1 (LRRC1) is upregulated in the bone marrow tissues of AML patients and AML cells, and lower overall survival was observed in patients with higher LRRC1 expression. The knockdown of LRRC1 inhibited cell cycle progression as well as glycolysis, and accelerated apoptosis, but these events can be rescued by MACF1 expression. LRRC1 depletion inhibited the xenograft tumor growth of HL-60 cells in nude mice, and downregulated MACF1 expression as well as β-catenin/c-Myc signaling in tumor tissues. In addition, LRRC1 can bind and regulate MACF1 expression. These data suggested that LRRC1 might activate β-catenin/c-Myc signaling to promote AML tumor growth by binding and regulating the expression of MACF1. Therefore, MACF is critical for AML tumor development [34].

### 2.2. Colorectal Cancer

As the third most common malignancy worldwide and the second leading cause of cancer-associated mortality, the incidence and mortality rates of CRC remain high, posing an urgent need to look for a novel way of treatment, in addition to the current therapeutics [56]. Kafaie et al. applied three algorithms, namely, BOolean Operation-based Screening and Testing (BOOST), FastEpistasis, and Tree-based Epistasis Association Mapping (TEAM), to genome-wide association studies (GWAS) data of colorectal cancer (CRC) to identify interacting genetic risk factors, and key genes such as *MACF1*, *USP49*, *SMAD2*, *SMAD3*, *TGFBR1*, *RHOA*, and *CCDC32* were highlighted. Thus, the pathological relevance of MACF1 to CRC is worthy of being confirmed and investigated [35].

### 2.3. Glioblastoma

Glioblastoma (GBM) is a lethal primary cancer of the brain [57]. Previous study has shown that MACF1 is substantially expressed in GBM tumor tissues instead of the low-grade oligodendroglioma, medulloblastoma, and the counterpart normal brain tissues. MACF1 is expressed in higher-grade brain tumors and in several tested GBM cell lines. MACF1 knockdown decreased cell proliferation in patient-derived GBM cell lines and cell migration in U251 GBM cells. Knocking down the expression of MACF1 and co-treatment with the chemotherapeutic drug temozolomide (TMZ) suppressed the cell proliferation of GBM cells, as compared to treatments with MACF1 siRNAs, control siRNAs, TMZ, or vehicle alone [58]. Furthermore, the same group developed a combinatorial radiotherapy for GBM by the co-treatment of GBM cells with MACF1 siRNAs and radiation. The results showed that MACF1 depletion sensitizes GBM cells to radiation, affecting cell viability and migration, and decreases the expression of p-ribosomal protein S6 of the mTOR signaling pathway, suggesting that this combinatorial strategy would be promising for GBM therapy and also highlighting the important role of MACF1 in GBM therapy [36].

### 2.4. Laryngeal Squamous Cell Carcinoma

Laryngeal squamous cell carcinoma (LSCC) is the most common histological subtype of laryngeal cancer, accounting for 85 % to 95 % of this disease [59]. A report revealed that circular MACF1 RNAs (circ_MACF1) were expressed substantially in laryngeal squamous cell carcinoma (LSCC) tissues, cells, and exosomes isolated from LSCC cells. MACF1 was positively regulated by circ_MACF1. Circ_MACF1 depletion inhibited LSCC-cell proliferative, migratory, and invasive activities through the suppression of PI3K/AKT/mTOR signaling to promote autophagy, which can be reversed by MACF1 overexpression. Exosomal circ_MACF1 was able to orchestrate PI3K/AKT/mTOR-mediated inhibition in autophagy to promote LSCC progression. Therefore, targeting LSCC through manipulating circ_MACF1 could be promising for LSCC therapy [37].

### 2.5. Lung Cancer

Lung cancer is among the top causes of cancer-associated deaths worldwide. More than 85% of lung cancer is non-small-cell lung cancer (NSCLC) and about 65% of NSCLC is lung adenocarcinoma (LUAD) [60]. Knowing the regulation leading to the pathogenesis of LUAD will be a workable way to combat this disease. Studies have shown that competing endogenous RNAs (ceRNAs) contain the same miRNA response elements (MREs) as RNA molecules. ceRNAs can bind miRNA and release the inhibition of miRNA to RNA molecules [61]. For example, the long non-coding RNA (lncRNA) DiGeorge syndrome critical region gene 5 (DGCR5) can function as a ceRNA to regulate LUAD progression by inhibiting miRNA hsamiR-22-3p [62]. A paper reported the role of long non-coding RNA (lncRNA)-associated competing endogenous RNA (ceRNA) in LUAD. Searching the Gene Expression Omnibus databases showed that the downregulation of the receptor activity modifying protein 2-anti-sense RNA 1 (RAMP2-AS1) was most prominent in LUAD, and microRNA (miR)-296-5p was identified as a binding target of RAMP2-AS1. Five genes—namely, *CD44*, cyclin D3 (*CCND3*), neurocalcin δ (*NCALD*), microtubule-actin cross-linking factor 1 (*MACF1*), and potassium channel tetramerization domain-containing 15 (*KCTD15*)—were isolated by intersecting the predicted target genes of miR-296-5p and 368 differentially expressed mRNAs in LUAD. Further analyses showed that these five genes were downregulated in LUAD, and their expression was positively correlated with that of RAMP2-AS1. Among these five genes, MACF1 expression was lower in tumor tissues as compared to that of normal tissue, while miR-296-5p expression was the opposite of MACF1. Higher MACF1 expression leads to higher overall survival (OS) and first-progression survival (FP) rates in patients with lung adenocarcinoma [38]. These data indicate that RAMP2-AS1, miR-296-5p, and MACF1 would be potential biomarkers of LUAD and their participation in LUAD pathogenesis is worthy of study.

It is known that circular RNA circ_MACF1 (circRNA ID: hsa_circ_0011780) can inhibit the proliferation and metastasis of non-small-cell lung cancer [63]. However, the role of circ_MACF1 in paclitaxel (PTX) resistance in lung adenocarcinoma (LUAD) remains to be determined. A study showed that circ_MACF1 expression was downregulated in PTX-resistant LUAD tissues and cells. Circ_MACF1 overexpression decreased proliferation, migration, and chemoresistance, and induced apoptosis in PTX-resistant LUAD cells, suggesting the potential use of circ_MACF1 for the treatment of LUAD [39].

### 2.6. Melanoma

Melanoma is the most malignant cancer of skin origin. Current treatment for melanoma includes surgery, radiation therapy, immunotherapy, targeted therapy, and systemic chemotherapy [64]. Despite the progress in these approaches, some drawbacks still remain that hinder effective treatment. A study reported that MACF1 knockdown decreased the colony formation and invasion activities of B16F10 melanoma cells. Tumor growth and lung metastasis were suppressed in MACF1 RNAi-B16F10-cell-injected mice. Further analyses showed that E-cadherin and Smad7 were increased while N-cadherin and TGF-β1 were decreased in the tumor tissues of the mice [40], suggesting that MACF1 may control metastasis through regulating the epithelial–mesenchymal transition, and thus, MACF1 could be a therapeutic target for the treatment of melanoma.

### 2.7. Muscle-Invasive Bladder Cancer (MIBC)

In bladder cancer (BLCA), the most common genitourinary malignancy—about 25%—is muscle-invasive bladder cancer (MIBC), which involves a high risk of metastasis. In addition to traditional therapy, anti-cancer immunotherapy is used for the treatment of MIBC. However, the objective response rate (ORR) is low due to the shortage of precise biomarkers for the selection of suitable patients for immunotherapy. For this purpose, scientists have developed novel MIBC immunotypes using clustering analysis based on the molecular subtype, tumor mutation burden (TMB), and CD8^+^ T cells. Three groups of subtypes have been defined, which are immunotype A, immunotype B, and immunotype C. Mutated genes have also been identified by several approaches and used as biomarkers to predict the specific immunotype. The results showed that seven genes—namely, *ARID1A*, *KDM6A*, *KMT2D*, *MUC16*, *TP53*, *SYNE1*, and *TTN*—displayed a high-frequency mutation rate in all immunotypes, while *RB1* (immunotypes A and C), *PIK3CA* (immunotypes A and C), *MACF1* (immunotype B), *FGFR3* (immunotype B), *KMT2C* (immunotype B), *RYR2* (immunotype A), and *EP300* (immunotype C) are differentially mutated among the three immunotypes. Survival analysis revealed that immunotype A had better overall survival (OS), and immunotype B had a medium OS, while immunotype C displayed a low OS. In addition, immunotype A had better treatment outcomes, immunotype B was associated with a low histologic grade, and immunotype C was linked to high tumor recurrence. Based on these studies, the three immunotypes can be applied to identify suitable MIBC patients for immunotherapy in a more precise way and, thus, increase the ORR during the treatment of bladder cancers [41].

### 2.8. Ovarian Cancer

Ovarian cancer remains the fifth leading cause of cancer-related deaths in women. Although systemic therapies have been applied to sustain the survival of patients, better methods for the treatment of advanced ovarian cancer to increase long-term survival and cure rates are pending [65]. A study reported the gene expression of MACF1 in fresh tumor tissues and the associated normal tissues of eighteen serous ovarian cancer (SOC) patients by quantitative RT-PCR, and the protein expression of MACF1 from paraffin-embedded SOC tissues and paratumor tissues by immunohistochemistry. The expression of MACF1 was higher in tumor tissues than in paratumor tissues. This high expression was positively correlated with shorter recurrence-free survival (RFS) and overall survival (OS) in SOC patients. Further analysis showed that the high expression of MACF1 is an independent poor survival predictor in SOC patients. These data suggest that MACF1 may be used as a prognostic biomarker for SOC [42].

Another laboratory reported a genetic study of patients with low-grade serous ovarian carcinoma (LGSOC) for mutation and copy-number variation by whole-exome sequencing, which identified 11% (8/71 cases) of patients with an *MACF1* mutation, and concluded that *MACF1* could be a novel driver gene for LGSOC [43].

### 2.9. Pancreatic Cancer

Pancreatic cancer (PaCa) is a fatal disease, the underlying mechanism and prognosis of which remain unsolved. A group of scientists employed the weighted gene co-expression network analysis (WGCNA) of the GSE21501 profile to search for potential genes that are linked to the overall survival (OS) events of PaCa patients. Some of the genes were selected for further Cox regression analysis, and a six-gene signature (*MACF1*, *FTSJ3*, *STAT1*, *STX2*, *CDX2*, and *RASSF4*) was identified that could successfully predict the overall survival (OS) of PaCa patients, which could be further validated in the TCGA-PAAD cohort. Their data suggest that this six-gene signature could be precisely applied to the prognosis of patients with pancreatic cancers [44].

### 2.10. Vulvar Squamous Cell Carcinoma (VSCC)

Vulvar squamous cell carcinoma (VSCC) is a rare disease that comprises two etiological pathways, which are the human papillomavirus (HPV)-dependent pathway and an HPV-independent pathway. By using whole-exome sequencing in 34 affiliated patients of this disease, scientists identified a total cohort of 1848 cancer-related mutations. Most of them were TP53 missense mutations (56%; 19/34), followed by HPV16-positive cases (35.3%; 12/34), which were mutually exclusive to TP53 mutations. Some less-frequent mutations of cancer-related genes were also discovered, including *MACF1* (14.7%; 5/34). Whether these mutations in MACF1 contribute to VSCC development, as well as its progression and subsequent underlying mechanisms, is worthy of further study [45].

## 3. Roles of MACF1 in Other Diseases

### 3.1. Alzheimer’s Disease

Alzheimer’s disease (AD) is the main form of dementia that typically affects older persons. There is no cure for AD so far, and the average survival time is 4 to 8 years from diagnosis [66]. As a cytolinker that might chelate all types of cytoskeleton, MACF1 is important in orchestrating cytoskeletal networks. Studies showed that GSK3 beta can phosphorylate on the MTBD of MACF1, leading to the dissociation of microtubules [67,68]. Other studies showed that GSK3 beta activity is de-regulated by binding to Aβ and its receptor PirB, probably through PP2A [69,70]. By collecting these and other evidence, scientists proposed a hypothesis to explain the possible involvement of MACF1 in the pathogenesis of Alzheimer’s disease (AD). It has been known that extracellular Aβ could be an important factor to trigger AD [71]. According to their hypothesis, the binding of Aβ to its membrane receptor—paired immunoglobulin-like receptor B (PirB)—activates cytosolic protein phosphatase 2A (PP2A), which in turn dephosphorylates and activates GSK3β. GSK3β then phosphorylates on the serine residue of the SRXXS region in the microtubule-binding domain (MTBD) of MACF1, resulting in a dissociation of MACF1 from microtubules, an alteration in cytoskeletal networks—and hence, the structure and function of synapses—and a reduction in plasticity, the loss of recognition, and finally AD. In vitro and in vivo experiments are urgently needed to prove this hypothesis [46].

### 3.2. Bipolar Disorder

Bipolar disorder, characterized by manic and depressive symptoms, is a mental disorder that affects approximately 1% of individuals [72]. Although efforts have been made, common variants discovered so far can only cover 25% of the heritability [73], suggesting that some other genetic factors leading to bipolar disorder need to be identified. Trio-based exome sequencing of a combined group with bipolar I and schizoaffective disorders for the enrichment of de novo, loss-of-function (LOF), or protein-altering mutations identified de novo mutations that were enriched in calcium-related genes. Two of them, *EHD1* and *MACF1*, were selected for further analyses. The *EHD1* patient had a truncation mutation and the *MACF1* patient had a frame-shift mutation (p.V266fs). The truncation mutation of *EHD1* resulted in reduced neurite outgrowth and inhibited endocytosis. Two knock-in mouse lines with these mutations generated by the CRISPR/Cas9 system were subjected to behavioral tests, including IntelliCage and long-term wheel running analyses. *Ehd1* mutant mice displayed higher activity in the light phase, while *Macf1* mutant mice exhibited a reduced attention span and reduced levels of persistence to reach rewards. These behaviors resemble those of the bipolar disorder animal model. The pathogenesis of bipolar disorder caused by the frame-shift mutations in MACF1 needs to be further explored [18].

### 3.3. Brain Malformation

A study has shown that de novo *MACF1* variants are linked to complex brain malformation. The mutation in the GAR domain of the MACF1 protein results in defects in neuronal migration and axon guidance [74]. On checking the brain magnetic resonance imaging of an infant with a pathogenic de novo *MACF1* variant [c.15682G>T p.(Asp5228Tyr)], lissencephaly with brainstem hypoplasia and dysplasia was observed. In addition to the brain malfunction, the infant displayed severe developmental delay, spasticity, and seizures within the first year [15]. These data suggest that MACF1 is important for neuronal activity and brain function.

### 3.4. Congenital Myasthenic Syndrome

The clinical and genetic characterization of a large Indian congenital myasthenic syndrome cohort by hotspot screening, followed by whole-exome sequencing in 156 genetically diagnosed patients from 141 families, identified *MACF1* as a novel candidate gene whose mutation is responsible for causing the neuromuscular junction defects of the disease [47].

### 3.5. Familial Psychosis

Previous study has shown that rare variants of the *MACF1* gene were identified in patients with schizophrenia (SCZ) [75]. Another group performed whole-exome sequencing in a Majorcan family enriched for psychosis, with two subjects with schizophrenia (SCZ) and two with schizoaffective disorder (SCA), to look for rare single-nucleotide variants (SNVs), SNPs, and copy-number variants (CNVs). The results showed that a missense mutation (g.39914279 C>G; T-4642-S) in the *MACF1* gene co-segregates with psychosis. Two CNVs, DUP3p26.3 and DUP16q23.3, were also identified in this family, which are reported to be able to affect the expression of two relevant genes, *CNTN6* and *CDH13*. The participation of MACF1, together with its interplay with CNTN6 and CDH13 in psychosis needs to be further elucidated [16].

### 3.6. The 1p34.2p34.3 Microdeletion Syndrome

MACF1 is located in chromosome 1p34.2–p34.3, and its microdeletion in this region (the 1p34.2p34.3 microdeletion syndrome) is linked to autism [48]. However, how MACF1 deletion affects neurodevelopment and brain function needs to be characterized. Another study reported the deletion of MACF1 in the developing mouse cerebral cortex, concentrating on radial glia polarity and morphological integrity. The researchers found that MACF1 deletion led to subcortical band heterotopia and disrupted cortical lamination during development. As compared to control cells, cell proliferation was pronounced in MACF1-deleted radial progenitors, which was associated with increased cell cycle speed and re-entry. A decrease in actin polymerization and microtubule stability along the apical ventricular area was observed in the MACF1 mutant cortex. A disconnection between radial glial fibers and the apical and pial surfaces was also observed. In addition, MACF1 deletion mutant mice displayed abnormal emotional behaviors and social deficits. These data outline the role of MACF1 in cortical progenitor proliferation and localization through supporting glial fiber stabilization and polarization [49].

### 3.7. Premature Ovarian Insufficiency

Ovarian deficiency, one of the main causes of female infertility, includes premature ovarian insufficiency (POI) and diminished ovarian reserve (DOR). The genetic factors leading to these defects are largely understudied. Scientists performed whole-exome sequencing (WES) in patients with ovarian deficiency and found the previously identified genes *STAG3*, *GDF9*, *FANCM*, and *FSHR* to be putative causative variant genes of POI. Novel candidate genes, including *XPO1*, *NRIP1*, and *MACF1*, were also identified. The involvement of *STAG3*, *GDF9*, *FANCM*, and *FSHR* in POI pathogenesis was revealed by functional experiments. Further analysis of the novel candidate genes in premature ovarian insufficiency, including *MACF1*, is pending [50].

### 3.8. Spectraplakinopathy

A study reported whole-exome sequencing in a family with spectraplakinopathy and found that both parents carried compound heterozygous missense mutations; the male parent carried the c.1517C>T (p. Thr506Ile) missense mutation and the female parent the c.11654T>C (p.Ile3885Thr) missense mutation in the *MACF1* gene. Although both parents were healthy, their two sons inherited both alleles of the mutations and developed neuromuscular disorders that present with progressive spastic tetraplegia, dystonia, joint contracture, feeding difficulty, and developmental delay. The Thr506Ile mutation occurs on a location between the LRR4 (repeated sequence motifs) and SH3 (Src homology 3) domains, and the Ile3885Thr mutation is located on the spectrin domain of MACF1, suggesting that regions covering these mutations harbor important biological functions [17].

### 3.9. Age-Related Osteoporosis

Age-related osteoporosis gains more and more attention with the aging of the human population. Two main factors, the decrease in bone formation and osteogenic differentiation, affect age-related osteoporosis. Previous studies have shown that the cytolinker protein MACF1 can promote the cell proliferation of osteogenic cells and regulate osteogenic differentiation and bone formation [76]. Another scientist explored the role of MACF1 in age-related osteoporosis. Western blotting and in situ hybridization analyses showed that the protein level of MACF1, as well as osteogenic markers *Ocn* (osteocalcin) and *Osterix,* was decreased in the femur tissues of the older group of patients with osteoporosis aged 80–95 (vs. the group aged 60–79) and in 21-month-old mice (vs. the group aged 6 months). The expression of MACF1 was positively correlated with the expression of *Ocn* and *Osterix.* These results indicated that MACF1 is associated with the bone formation reduction in the elderly population. The protein expression of MACF1 and osteogenic differentiation were substantially reduced in bone marrow mesenchymal stem cells (BMSCs) derived from aging mice (21-month-old mice). The protein level of hairy/enhancer of split 1 (HES1), the transcription factor that was implicated in the inhibition of bone formation [77] and osteogenic differentiation [78], was upregulated in the femur tissues of the older group of patients with osteoporosis aged 80–95 (vs. the group aged 60–79) and in 21-month-old mice (vs. the group aged 6 months). The ectopic expression of MACF1 decreased the protein expression and activity of HES1, while the knockdown of MACF1 increased the protein expression and activity of HES1 in MC3T3-E1 osteoblastic cells. These data suggest that MACF1 is able to support bone formation by suppressing the activities of HES1 in aging-related osteoporosis. The in situ injection of MACF1 plasmid showed that the forced expression of MACF1 can rescue aging-related osteoporosis and post-menopausal osteoporosis. These data clearly point out the function of MACF1 in bone formation and osteogenic differentiation and open an avenue for curing aging-related osteoporosis through the manipulation of MACF1 [51].

The decrease in osteogenic potential in mesenchymal stem cells (MSCs) contributes to senile osteoporosis (SOP). The downregulation of Wnt/β-catenin signaling in MSCs is one of the causes of SOP. As MACF1 is an important Wnt/β-catenin regulator [12], MSC-specific Prrx1 (Prx1) promoter-driven MACF1 conditional knock-in (MACF-KI) mice were generated using naturally aged male mice and ovariectomized female mice models in order to study the effects and underlying mechanisms of the ectopic expression of MACF1 in MSCs on the regulation of SOP. The results showed that the forced expression of MACF1 can fortify MSC osteogenesis and bone formation via the TCF4/miR-335-5p signaling pathway in SOP, indicating that MACF1 could be a novel therapeutic target for the treatment of SOP to increase bone formation [52].

### 3.10. Diabetic Osteoporosis

Misashi 1 (MSI1) is an RNA-binding protein that can target protein mRNA to regulate its protein expression, and is believed to be involved in osteogenic differentiation [79]. RNA immunoprecipitation revealed that MSI1 can bind to MACF1 mRNA molecules. Murine osteoblastic MC3T3-E1 cells were challenged with high glucose (HG; 30mM) to mimic diabetic osteoporosis in vivo. The HG treatment inhibited cell proliferation, promoted cell apoptosis, and suppressed osteogenic differentiation. The expression of both MSI1 and MACF1 was decreased when the MC3T3-E1 cells were challenged with HG. The forced expression of MSI1 accelerated cell proliferation and osteogenic differentiation, while inhibiting apoptosis in the HG-challenged MC3T3-E1 cells, although these activities could be reversed by MACF1 depletion. In addition, MSI1 overexpression increased MACF1 expression. Therefore, MSI1 upregulation stabilizes MACF1 to inhibit apoptosis and stimulate osteogenic differentiation in HG-challenged MC3T3-E1 cells. This study also highlights the importance of MACF1 in diabetic osteoporosis [53].

### 3.11. Proliferative Diabetic Retinopathy

It is known that RNA-binding proteins (RBPs) can regulate gene expression via alternative splicing events (ASEs), contributing to the pathogenesis of proliferative diabetic retinopathy (PDR), a disease that involves retinal neovascularization (RNV) and vision loss in diabetic patients. Scientists searched public transcriptome datasets of PDR and normal retinas to reveal the differentially expressed genes between these two samples. Selected differentially expressed RBPs were chosen for the screening of co-expressed alternative splicing genes (ASGs)—and one of the enriched pathways is the cell cycle pathway—using a functional assay. Then, a cell cycle-linked ASE and an RBP-AS regulatory network were established and extracted, and one of the confirmed targets in the mouse model of oxygen-induced retinopathy was MACF1. MACF1 theoretically functions as an RNA-binding protein that regulates the alternative splicing of cell cycle-associated genes in proliferative diabetic retinopathy, and will therefore be a promising treatment target for the disease [54].

## 4. Physiological Roles of MACF1

### 4.1. Adhesome Architecture

An earlier study showed that the knockdown of integrin αV in MDA-MB-435S melanoma cells decreased cell migration and sensitized cells to paclitaxel and vincristine, two microtubule (MT) poisons [80]. Proteomics analyses of integrin αV-knockdown MDA-MB-435S cells by mass spectrometry (MS) revealed that key components of the integrin αVβ5 adhesion complexes—including talins 1 and 2, α-actinins 1 and 4, filamins A and B, plectin, and vinculin—were decreased compared to naive MDA-MB-435S cells. Several components of the cortical microtubule stabilization complex that bridges MTs to adhesion sites, especially liprins α and β, ELKS, LL5β, KANK1, KANK2, and MACF1, were also decreased. MDA-MB-435S cells that knocked down KANK2 mimicked the integrin αV-knockdown MDA-MB-435S cells in levels of migration and sensitivity to MT poisons. These data suggest that KANK2 orchestrates αVβ5 focal adhesions with microtubules to control cell migration and sensitivity to microtubule poisons [19]. The functional roles of MACF1 in adhesion sites could be an interesting area for further research.

Mitofusin-2 (Mfn2) is a GTPase that is located on the outer mitochondrial membrane and participates in mitochondrial fusion [81]. By using three-dimensional structured illumination microscopy, scientists reported that Mfn2 colocalizes with VE-cadherin and beta-catenin at the plasma membrane and plays a role in stabilizing the endothelial barrier during homeostasis in human lung microvascular endothelial cells (HLMVECs). Upon inflammatory stimulation, the Mfn2/beta-catenin complex was released from adherens junctions (AJs), and Mfn2 could accumulate in the nucleus to suppress the transcriptional activity of beta-catenin. The endothelial-specific abscission of Mfn2 resulted in the disruption of filamentous actin and the activation of inflammation, suggesting the role of Mfn2 in suppressing the inflammatory response by stabilizing the cell–cell adherens junctions (AJs). Binding partners of GFP-Mfn2 in HLMVECs were identified by high-affinity liquid chromatography followed by tandem mass spectrometry (LC-MS/MS). In addition to mitochondrial binding partners, cadherin-binding partners and cell–cell adhesion partners such as KRT18, ENO1, and MACF1 were retrieved. The functional interaction of MACF1 with Mfn2 can be further studied [20].

Focal adhesions (FAs) can link the cellular infrastructure to the extracellular matrix (ECM) to regulate cell adhesion, migration, and mechanosensing. It is known that FAs contain three vertical layers, namely, an ‘actin-regulatory layer’, a ‘force-transduction layer’, and an ‘integrin signaling layer’, which connect the cytoskeleton to the ECM. Scientists employed super-resolution interferometric photo-activated localization microscopy (iPALM), together with TIRF microscopy, and discovered two novel nanoscale layers within FAs, which organize actin filaments to tropomyosin (Tpm) isoforms Tpm1.6 and Tpm3.2, with Tpm1.6-actin filaments located on the dorsal side and Tpm3.2-actin filaments on the ventral side of the FAs. Tpm3.2 stabilizes ACF7/MACF1 and KANK-family proteins at the FAs, and thus targets MT-plus-ends to FA to catalyze FA disassembly. These data indicate that FAs contain multiple actin filament layers to regulate FA dynamics by linking to the cytoskeleton [21].

### 4.2. Bone Formation

Previous studies have indicated that MACF1 is important for the proliferation, cell cycle progression, and differentiation of murine preosteoblast MC3T3-E1 cells [82] [76,83,84]. Osteoblast-specific Osterix (Osx)-promoter-driven MACF1 conditional knockout mice (*Macf1*^f/f^ Osx_-_Cre) were generated to further study the role of MACF1 in bone formation. The mutant mice showed delayed ossification and reduced bone mass, with weakened biomechanical femur strength. Furthermore, retarded differentiation, a reduced expression of osteogenic marker genes (*Col1*, *Runx2*, and *Alp*), a reduced number of mineralized nodules, and decreased Bmp2/Smad/Runx2 signaling were observed in primary osteoblasts of mutant mice. The underlying mechanism of how MACF1 is involved in bone formation through Bmp2/Smad/Runx2 signaling needs to be further studied [22].

Another group of scientists discovered that *MACF1* expression is reduced in mesenchymal stem cells (MSCs) of osteoporotic bones. The conditional knockout of MACF1 in mesenchymal tissues resulted in ossification retardation in the skull and hindlimb of young mice and a reduction in bone mass, bone microarchitecture, and bone formation ability in adult mice. Further evidence showed that MACF1 can bind SMAD family member 7 (SMAD7) and shuttle SMAD7 into the nucleus to induce downstream osteogenic pathways. This study highlights the role of MACF1 in osteogenesis through binding and transporting SMAD7 into the nucleus [85].

A previous study indicated that miRNA miR-138-5p can function as a mechanosensitive miRNA in osteogenic differentiation [86]. Another study showed that miR-138-5p was upregulated in the bone tissues of bedridden and aged patients that suffered from a lower degree of bone formation. Further studies showed that MACF1 is a direct target of miR-138-5p. The targeted inhibition of miR-138-5p in the bone increased the mechanical bone anabolic response in the hindlimb of unloaded mice and sensitized the bone anabolic response to mechanical loading in aged mice and miR-138-5p transgenic mice to support bone formation. These data indicate that the depletion of mechanosensitive miR-138-5p can increase bone anabolic activity in response to mechanical stimuli, probably through upregulating MACF1 expression [23].

### 4.3. Neuronal Aging

Scientists established a new cellular model within the Drosophila brain based on the characterization of neuronal aging in cells positioned within the fly’s visual system. Distinctive aging hallmarks, including axonal swellings, altered axon diameters, and cytoskeletal and synaptic decay, are also observed in mammals. The reduction in the expression of three microtubule (MT) regulators, namely, MACF1-homolog short stop (shot), Tau, and end-binding protein 1 (EB1), elicited aging-like MT catastrophe and worsened age-dependent neuronal decay. The expression of these three MT regulators compromised these effects. These data outline the importance of these microtubule-binding proteins, including MACF1-homolog short stop, in neuronal aging [24].

### 4.4. Osteoblast Differentiation

It has been known that MACF1 is involved in osteoblast differentiation; however, the underlying mechanism remains unresolved. Scientists studied the role of MACF1 in osteoblast differentiation and discovered that MACF1 can regulate the cytoplasm–nucleus shuttling of itself and two-binding partners, transcription factor 12 (TCF12) and E2F transcription factor 6 (E2F6), two repressors of osteoblast differentiation. A high amount of MACF1 can sequester TCF12 and E2F6 in the cytoplasm and thus promote osteoblast differentiation [25]. These data suggest that MACF1 displays a novel role by participating in osteoblast differentiation through trapping the repressors of transcription factors in the cytoplasm.

### 4.5. Preosteoblast Migration

Preosteoblast migration is important for bone mass maintenance [87]. MACF1-overexpressed and knockdown stable cell lines of MC3T3-E1 cells were derived to study the role of MACF1 in preosteoblast migration. Both in vitro and in vivo experiments showed that the overexpression of MACF1 promotes preosteoblast migration, while MACF1 reduction decreases it. Further experiments showed that MACF1 overexpression activates Src to phosphorylate end-binding protein 1 (EB1), which reduced the association of EB1 with adenomatous polyposis coli (APC) in focal adhesion, leading to the release of EB1 from the focal adhesion complex and the re-distribution of EB1 on microtubules, and thus the promotion in FA turnover and preosteoblast migration. These data suggest that MACF1 could be a good candidate for the treatment of bone-related diseases [26].

### 4.6. Tooth Development

Osterix/Sp7 is a zinc-finger bone-related transcription factor that participates in osteoblast differentiation and bone maturation [88]. Osterix (Osx) is expressed in dental mesenchyme during tooth development [27]. A paper reported the generation of *Osx-Cre:Macf1^f/f^* mice that specifically delete MACF1 in odontoblasts for a tooth morphology study. *Macf1* deprivation in odontoblasts deteriorated tooth morphology and microarchitecture in both dentin and enamel. Both odontoblast differentiation and ameloblast differentiation were affected by MACF1 deficiency in odontoblasts through the reduced secretion of IGF1 by odontoblasts [27].

## 5. Other Roles of MACF1

### 5.1. MACF1 as a Biomarker for Predicting Bloodstream Infections in Children with Burns

The least absolute shrinkage and selection operator (LASSO) machine learning algorithm was used to select a panel of biomarkers predictive of bloodstream infections (BSI) as the outcome of burned patients (total burn surface area (TBSA) ≥20%) by analyzing their blood transcriptome. This search revealed a panel of ten probe sets corresponding to six annotated genes (*ARG2*, *CPT1A*, *FYB*, *ITCH*, *MACF1*, and *SSH2*) and two uncharacterized *(LOC101928635* and *LOC101929599*) and two unannotated regions, which can be used as markers with a high prediction rate. Prediction using these biomarkers, including *MACF1*, will help with suitable treatments before infection occurs [28].

### 5.2. MACF1 SNP Is Associated with Eyebrow Thickness in the Male Pakistani Punjabi Population

Sixteen single-nucleotide polymorphisms (SNPs) in 58 male individuals from the Punjabi population of Pakistan were obtained to link multiple genetic variants at distant loci that are associated with facial hair traits, including eyebrow thickness, beard thickness, and monobrow. Among these SNPs, *rs6684877* in *MACF1* exhibited a significant association with eyebrow thickness, *rs365060* in *EDAR* and *rs12597422* in *FTO* displayed a strong association with monobrow, while two other SNPs (*rs9654415* and *rs7702331*) in *LOC105379031* were highly linked to beard thickness. This study indicated that MACF1 can be used for forensic molecular photofitting in the future [29].

### 5.3. MACF1 Serves as a Genetic Factor for Selection Signatures

To build up selection signatures of Fuzhong Buffalo, an important animal for the Guangxi Zhuang Autonomous Region of China, scientists analyzed 27 whole genomes of buffalo and used several approaches to select candidate genes in pathways that are involved in response to exercise, immunity, and the nervous system. Some genes that are related to product and growth traits were also identified, including *MACF1*. These genes provide the basis for future research on the selection and breeding of buffalo [30].

Genome-wide association studies (GWAS) were used to retrieve genomic information that is linked to the body-weight trait of 14-month-old Alpine Merino Sheep, and eight candidate genes that affect live weight were identified—namely, *FAM184B*, *NCAPG*, *MACF1*, *ANKRD44*, *DCAF16*, *FUK*, *LCORL*, and *SYN3*—which are believed to be involved in muscle growth and bone formation in sheep. This result indicates that MACF1 could serve as a marker for genome selection breeding [31].

### 5.4. Correlation of DNA Methylation Patterns of MACF1 Genes with Bull Offspring Development

Bulls of the Pacific Northwest region of the United States and other northern regions suffer from extreme cold temperatures during winter. The impact of cold weather on the methylation patterns in the sperm of the same bull collected during late spring and winter was studied by whole-genome bisulfite sequencing (WGBS). In total, 3165 differentially methylated cytosines (DMCs) were identified in 438 differentially methylated regions (DMRs) across 186 genes induced by cold shock. Eight unique differentially methylated genes (DMGs), including *MACF1*, involved in embryonic development and nine unique DMGs involved in osteogenesis were revealed, and eight of these DMGs were confirmed by methylation-specific PCR (MS-PCR). These DMGs, including *MACF1*, can be applied in the evaluation of bull offspring development in the future [32].

## 6. Concluding Remarks and Future Perspectives

MACF1 is a titanic protein and its multifaceted functions come from its multiple-domain nature (Figure 1). MACF1a can bind actin filaments and microtubules and function as a linker between these two elements. In addition to linking actin filaments and microtubules, MACF1b is believed to coordinate all three cytoskeletal elements due to its additional conserved plectin-repeat (plakin-repeat) domain (PRD) for potential binding to intermediate filaments (Figure 1) [7], although this latter activity needs to be further proved. To cope with its nature of multiple functions, MACF1 is located on the cell membrane [89] and shuttles between the cytoplasm and nucleus to execute its biological functions [25,85]. MACF1 participates in Wnt signaling as revealed by MACF1 knockout mice that display defects in the formation of mesoderm, node, and primitive streak, inducing mouse embryonic lethality [12]. This phenotype is similar to those of the Wnt 3 deletion [90] and LRP5/6 double-deletion mice [91], reinforcing the participation of MACF1 in the Wnt signaling pathway.

Given its multi-domain nature, we expect that there are more functions of MACF1 waiting to be discovered. One way to reveal new functions of MACF1 is to investigate the associated functions of the proteins that it interacts with by coupling co-immunoprecipitation or tandem-affinity purification with mass spectrometry analyses. MACF1 interactome network analyses can also be achieved using protein interaction databases such as BioGRID and STRING. Another way is to screen for natural mutations that create the phenotypic defects that disclose the potential functions of MACF1. Mutation in the MTBD causes lissencephaly (M1, Table 1). Mutation in spectrin repeats results in familial psychosis (M2, Table 1), while a combination of mutations in the plakin domain and spectrin repeats (M3 and M4 in Table 1) produce spectraplakinopathy. A frame-shift mutation of MACF1 (M5, Table 1) is linked to bipolar disorder [18]. Previous studies have also shown that mutations in different regions of MACF1 can cause a variety of symptoms, including type 2 diabetes, fetal akinesia, autism, and schizophrenia. The duplication and splicing variants of MACF1 can cause myopathy and craniosynostosis (Table I in [17]). Although these phenotypic defects in MACF1 mutants reflect the possible functions of MACF1, the underlying mechanisms that lead to these phenotypes need to be further addressed.

In addition to its physiological and pathological roles, MACF1 works as a genetic factor that is responsible for specific traits. The search for selection signatures for genes such as MACF1 that are involved in growth and product traits of Fuzhong Buffalo is an example [30]. MACF1 also serves as a marker for genome selection breeding [31]. As a differentially methylated gene, MACF1 can be applied in the evaluation of bull offspring perspective development [32]. It could also be used as a biomarker for predicting bloodstream infections in children with burns and help with suitable treatments before infection occurs [28]. In addition, SNPs including MACF1 are linked to facial features such as eyebrow thickness, which is useful for forensic molecular photofitting [29]. These studies reinforce the multifaceted role of MACF1.

Although a great deal of progress has been made in uncovering novel functions of MACF1, there are unresolved questions regarding this protein that remain to be addressed in the future: (1) The uncultivated regions of MACF1 are very likely to harbor specific biological activities for cellular processes, which remain to be discovered. (2) The plakin-repeat domain (or plectin-repeat domain) of the cytolinker protein plectin is known to bind intermediate filaments [92]. As the lengthy member, MACF1b also contains conserved PRDs (Figure 1), whether the PRD of MACF1b can bind and organize intermediate filaments for executing unknown biological functions is worthy of study. (3) There is evidence for the participation of MACF1 in various cancers [33,34,35,36,38,39,40,41,42,43,44,45] and a few other diseases [15,16,18,46,47,49,50,53,93]; however, how MACF1 and its interactors are involved in these diseases remains undetermined. Resolving the underlying mechanisms will help with the treatment of these diseases. (4) The downregulation of MACF1 gene expression [17,51,52] or loss-of-function mutations in the MACF1 gene (Table 1) lead to severe disorders. For example, a decrease in MACF1 levels results in age-related osteoporosis [51] and senile osteoporosis [52]. The tissue-specific expression of MACF1 achieved by the in situ injection [51] of MACF1 expression plasmids for MACF1-downregulated patients and the rescue of *MACF1* gene mutations using the CRISPR-Cas9 system would be possible ways to cure diseases that are caused by defects in MACF1. (5) MACF1 is known to be involved in various types of cancers (Figure 4). Specifically, it plays a positive role in the development of AML [34], GBM [36,58], LSCC [37], melanoma [40], and SOC [42], and a negative role in LUAD [38] and PTX-resistant LUAD [39], probably in a context-dependent manner or involving the microenvironment; the exact mechanisms need further exploration. (6) Bio-informatics and genomic analyses discovered the correlation between MACF1 and cancers, including CRC [35], LGSOC [43], and VSCC [45], which needs further experimental verification and mechanistic studies. Discovering the detailed mechanisms of MACF1 in cancer development will help with the subsequent treatment of the disease. 

In conclusion, accumulating evidence indicates that MACF1 is involved in many types of cancer (Figure 4) [33,34,35,36,37,38,40,41,42,43,44,45]. Studies also indicate that MACF1 plays important roles in a variety of other diseases and engages in multiple physiological activities (see the illustrations in Figure 2, Figure 3, and Figure 5). Therefore, MACF1 is a bona fide versatile giant. We believe that more functions of MACF1 remain to be explored and determined. Hopefully, this review will elicit more research on this multifaceted protein and can pave the way for the treatment of diseases caused by MACF1 defects.

## Abbreviations

MACF1: microtubule-actin cross-linking factor 1; ACF7: actin cross-linking factor 7; MTBD: microtubule-binding domain; GSK3 beta: glycogen synthase kinase 3 beta; CRC: colorectal cancer; LUAD: lung adenocarcinoma; NSCLC: non-small-cell lung cancer; ceRNA: competing endogenous RNA; circRNA: circular RNA; RAMP2-AS1: receptor activity modifying protein 2-anti-sense RNA 1; MIBC: muscle-invasive bladder cancer; BLCA: bladder cancer; SOC: serous ovarian cancer; OS: overall survival; RFS: recurrence-free survival; FP: first-progression survival; ORR: object response rate; PaCa: pancreatic cancer; VSCC: vulvar squamous cell carcinoma; AD: Alzheimer’s disease; PirB: paired immunoglobulin-like receptor B; PP2A: protein phosphatase 2A; POI: premature ovarian insufficiency; DOR: diminished ovarian reserve; HES1: hairy/enhancer of split 1; HLMVECs: human lung microvascular endothelial cells; MSCs: mesenchymal stem cells; TCF12: transcription factor 12; E2F6: E2F transcription factor 6.

## References

[1] Kodama A., Karakesisoglou I., Wong E., Vaezi A., Fuchs E. (2003). ACF7: An essential integrator of microtubule dynamics. Cell.

[2] Zhang J., Yue J., Wu X. (2017). Spectraplakin family proteins-cytoskeletal crosslinkers with versatile roles. J. Cell Sci..

[3] Suozzi K.C., Wu X., Fuchs E. (2012). Spectraplakins: Master orchestrators of cytoskeletal dynamics. J. Cell Biol..

[4] Cusseddu R., Robert A., Cote J.F. (2021). Strength Through Unity: The Power of the Mega-Scaffold MACF1. Front. Cell Dev. Biol..

[5] Sonnenberg A., Liem R.K. (2007). Plakins in development and disease. Exp. Cell Res..

[6] Jefferson J.J., Leung C.L., Liem R.K. (2004). Plakins: Goliaths that link cell junctions and the cytoskeleton. Nat. Rev. Mol. Cell Biol..

[7] Lin C.M., Chen H.J., Leung C.L., Parry D.A., Liem R.K. (2005). Microtubule actin crosslinking factor 1b: A novel plakin that localizes to the Golgi complex. J. Cell Sci..

[8] Skipworth R.J., Stewart G.D., Bhana M., Christie J., Sturgeon C.M., Guttridge D.C., Cronshaw A.D., Fearon K.C., Ross J.A. (2010). Mass spectrometric detection of candidate protein biomarkers of cancer cachexia in human urine. Int. J. Oncol..

[9] Burgo A., Proux-Gillardeaux V., Sotirakis E., Bun P., Casano A., Verraes A., Liem R.K., Formstecher E., Coppey-Moisan M., Galli T. (2012). A molecular network for the transport of the TI-VAMP/VAMP7 vesicles from cell center to periphery. Dev. Cell.

[10] Kakinuma T., Ichikawa H., Tsukada Y., Nakamura T., Toh B.H. (2004). Interaction between p230 and MACF1 is associated with transport of a glycosyl phosphatidyl inositol-anchored protein from the Golgi to the cell periphery. Exp. Cell Res..

[11] Ka M., Kim W.Y. (2016). Microtubule-Actin Crosslinking Factor 1 Is Required for Dendritic Arborization and Axon Outgrowth in the Developing Brain. Mol. Neurobiol..

[12] Chen H.J., Lin C.M., Lin C.S., Perez-Olle R., Leung C.L., Liem R.K. (2006). The role of microtubule actin cross-linking factor 1 (MACF1) in the Wnt signaling pathway. Genes Dev..

[13] Moffat J.J., Ka M., Jung E.M., Smith A.L., Kim W.Y. (2017). The role of MACF1 in nervous system development and maintenance. Semin. Cell Dev. Biol..

[14] Miao Z., Ali A., Hu L., Zhao F., Yin C., Chen C., Yang T., Qian A. (2017). Microtubule actin cross-linking factor 1, a novel potential target in cancer. Cancer Sci..

[15] Bolsterli B.K., Steindl K., Kottke R., Steinfeld R., Boltshauser E. (2021). Lissencephaly with Brainstem Hypoplasia and Dysplasia: Think MACF1. Neuropediatrics.

[16] Pol-Fuster J., Canellas F., Ruiz-Guerra L., Medina-Dols A., Bisbal-Carrio B., Asensio V., Ortega-Vila B., Marzese D., Vidal C., Santos C. (2021). Familial Psychosis Associated with a Missense Mutation at MACF1 Gene Combined with the Rare Duplications DUP3p26.3 and DUP16q23.3, Affecting the CNTN6 and CDH13 Genes. Front. Genet..

[17] Kang L., Liu Y., Jin Y., Li M., Song J., Zhang Y., Zhang Y., Yang Y. (2019). Mutations of MACF1, Encoding Microtubule-Actin Crosslinking-Factor 1, Cause Spectraplakinopathy. Front. Neurol..

[18] Nakamura T., Nakajima K., Kobayashi Y., Itohara S., Kasahara T., Tsuboi T., Kato T. (2021). Functional and behavioral effects of de novo mutations in calcium-related genes in patients with bipolar disorder. Hum. Mol. Genet..

[19] Paradzik M., Humphries J.D., Stojanovic N., Nestic D., Majhen D., Dekanic A., Samarzija I., Sedda D., Weber I., Humphries M.J. (2020). KANK2 Links αVβ5 Focal Adhesions to Microtubules and Regulates Sensitivity to Microtubule Poisons and Cell Migration. Front. Cell Dev. Biol..

[20] Kim Y.M., Krantz S., Jambusaria A., Toth P.T., Moon H.G., Gunarathna I., Park G.Y., Rehman J. (2021). Mitofusin-2 stabilizes adherens junctions and suppresses endothelial inflammation via modulation of β-catenin signaling. Nat. Commun..

[21] Kumari R., Ven K., Chastney M., Kokate S.B., Peranen J., Aaron J., Kogan K., Almeida-Souza L., Kremneva E., Poincloux R. (2024). Focal adhesions contain three specialized actin nanoscale layers. Nat. Commun..

[22] Qiu W.X., Ma X.L., Lin X., Zhao F., Li D.J., Chen Z.H., Zhang K.W., Zhang R., Wang P., Xiao Y.Y. (2020). Deficiency of Macf1 in osterix expressing cells decreases bone formation by Bmp2/Smad/Runx2 pathway. J. Cell. Mol. Med..

[23] Chen Z., Zhao F., Liang C., Hu L., Li D., Zhang Y., Yin C., Chen L., Wang L., Lin X. (2020). Silencing of miR-138-5p sensitizes bone anabolic action to mechanical stimuli. Theranostics.

[24] Okenve-Ramos P., Gosling R., Chojnowska-Monga M., Gupta K., Shields S., Alhadyian H., Collie C., Gregory E., Sanchez-Soriano N. (2024). Neuronal ageing is promoted by the decay of the microtubule cytoskeleton. PLoS Biol..

[25] Hu L., Yin C., Chen D., Wu Z., Liang S., Zhang Y., Huang Z., Liu S., Xu X., Chen Z. (2021). MACF1 promotes osteoblast differentiation by sequestering repressors in cytoplasm. Cell Death Differ..

[26] Su P., Yin C., Li D., Yang C., Wang X., Pei J., Tian Y., Qian A. (2020). MACF1 promotes preosteoblast migration by mediating focal adhesion turnover through EB1. Biol. Open.

[27] Qiu W., Lin X., Yang S., Chen Z., Zhang K., Yang C., Li Y., Miao Z., Deng X., Duan X. (2024). MACF1 deficiency suppresses tooth mineralization through IGF1 mediated crosstalk between odontoblasts and ameloblasts. Genes Dis..

[28] Tsurumi A., Flaherty P.J., Que Y.A., Ryan C.M., Banerjee A., Chakraborty A., Almpani M., Shankar M., Goverman J., Schulz J.T. (2023). A Preventive Tool for Predicting Bloodstream Infections in Children with Burns. Shock.

[29] Jawad M., Adnan A., Rehman R.A., Nazir S., Adeyemo O.A., Amer S.A.M., Hadi S., Liu F., Wang C.C., Rakha A. (2023). Evaluation of facial hair-associated SNPs: A pilot study on male Pakistani Punjabi population. Forensic Sci. Med. Pathol..

[30] Sun T., Huang G.Y., Wang Z.H., Teng S.H., Cao Y.H., Sun J.L., Hanif Q., Chen N.B., Lei C.Z., Liao Y.Y. (2020). Selection signatures of Fuzhong Buffalo based on whole-genome sequences. BMC Genom..

[31] Li C., Li J., Wang H., Zhang R., An X., Yuan C., Guo T., Yue Y. (2023). Genomic Selection for Live Weight in the 14th Month in Alpine Merino Sheep Combining GWAS Information. Animals.

[32] Hossain M.N., Gao Y., Hatfield M.J., de Avila J.M., McClure M.C., Du M. (2024). Cold exposure impacts DNA methylation patterns in cattle sperm. Front. Genet..

[33] Wang P., Zhang J., Zhang H., Zhang F. (2023). The role of MACF1 on acute myeloid leukemia cell proliferation is involved in Runx2-targeted PI3K/Akt signaling. Mol. Cell. Biochem..

[34] Wang Y., Tong H., Wang J., Hu L., Huang Z. (2024). LRRC1 knockdown downregulates MACF1 to inhibit the malignant progression of acute myeloid leukemia by inactivating β-catenin/c-Myc signaling. J. Mol. Histol..

[35] Kafaie S., Xu L., Hu T. (2021). Statistical methods with exhaustive search in the identification of gene-gene interactions for colorectal cancer. Genet. Epidemiol..

[36] Bonner K., Borlay D., Kutten O., Quick Q.A. (2020). Inhibition of the Spectraplakin Protein Microtubule Actin Crosslinking Factor 1 Sensitizes Glioblastomas to Radiation. Brain Tumor Res. Treat..

[37] Zhu Y., Duan C., Gui Y., Chen D., Su X. (2024). Exosomal circMACF1 drives PI3K/AKT/mTOR-mediated autophagy suppression in laryngeal squamous cell carcinoma. Cell. Mol. Biol..

[38] Song Z., Zhang Y., Chen Z., Zhang B. (2021). Identification of key genes in lung adenocarcinoma based on a competing endogenous RNA network. Oncol. Lett..

[39] Qian X., Chen C., Tong S., Zhang J. (2023). Circ_MACF1 targets miR-421 to upregulate FMO2 to suppress paclitaxel resistance and malignant cellular behaviors in lung adenocarcinoma. Thorac. Cancer.

[40] Wang X., Jian X., Dou J., Wei Z., Zhao F. (2020). Decreasing Microtubule Actin Cross-Linking Factor 1 Inhibits Melanoma Metastasis by Decreasing Epithelial to Mesenchymal Transition. Cancer Manag. Res..

[41] Chen Z., Liu G., Liu G., Bolkov M.A., Shinwari K., Tuzankina I.A., Chereshnev V.A., Wang Z. (2021). Defining muscle-invasive bladder cancer immunotypes by introducing tumor mutation burden, CD8+ T cells, and molecular subtypes. Hereditas.

[42] Liu L., Hu K., Zeng Z., Xu C., Lv J., Lin Z., Wen B. (2021). Expression and Clinical Significance of Microtubule-Actin Cross-Linking Factor 1 in Serous Ovarian Cancer. Recent Pat. Anticancer Drug Discov..

[43] Cheasley D., Nigam A., Zethoven M., Hunter S., Etemadmoghadam D., Semple T., Allan P., Carey M.S., Fernandez M.L., Dawson A. (2021). Genomic analysis of low-grade serous ovarian carcinoma to identify key drivers and therapeutic vulnerabilities. J. Pathol..

[44] Yang J., Shi W., Zhu S., Yang C. (2020). Construction of a 6-gene prognostic signature to assess prognosis of patients with pancreatic cancer. Medicine.

[45] Prieske K., Alawi M., Oliveira-Ferrer L., Jaeger A., Eylmann K., Burandt E., Schmalfeldt B., Joosse S.A., Woelber L. (2020). Genomic characterization of vulvar squamous cell carcinoma. Gynecol. Oncol..

[46] Wang X., Qi Y., Zhou X., Zhang G., Fu C. (2020). Corrigendum to ‘Alteration of scaffold: Possible role of MACF1 in Alzheimer’s disease pathogenesis’ [Med. Hypoth. 130 (2019) 109259]. Med. Hypotheses.

[47] Polavarapu K., Sunitha B., Topf A., Preethish-Kumar V., Thompson R., Vengalil S., Nashi S., Bardhan M., Sanka S.B., Huddar A. (2024). Clinical and genetic characterisation of a large Indian congenital myasthenic syndrome cohort. Brain.

[48] Kumar R.A., Sudi J., Babatz T.D., Brune C.W., Oswald D., Yen M., Nowak N.J., Cook E.H., Christian S.L., Dobyns W.B. (2010). A de novo 1p34.2 microdeletion identifies the synaptic vesicle gene RIMS3 as a novel candidate for autism. J. Med. Genet..

[49] Ka M., Moffat J.J., Kim W.Y. (2022). MACF1, Involved in the 1p34.2p34.3 Microdeletion Syndrome, is Essential in Cortical Progenitor Polarity and Brain Integrity. Cell Mol. Neurobiol..

[50] Jaillard S., Bell K., Akloul L., Walton K., McElreavy K., Stocker W.A., Beaumont M., Harrisson C., Jaaskelainen T., Palvimo J.J. (2020). New insights into the genetic basis of premature ovarian insufficiency: Novel causative variants and candidate genes revealed by genomic sequencing. Maturitas.

[51] Yin C., Tian Y., Hu L., Yu Y., Wu Z., Zhang Y., Wang X., Miao Z., Qian A. (2021). MACF1 alleviates aging-related osteoporosis via HES1. J. Cell. Mol. Med..

[52] Zhang K., Qiu W., Li H., Li J., Wang P., Chen Z., Lin X., Qian A. (2023). MACF1 overexpression in BMSCs alleviates senile osteoporosis in mice through TCF4/miR-335-5p signaling pathway. J. Orthop. Translat..

[53] Zou L., Xiang C., Lu M. (2023). MSI1 Stabilizes MACF1 to Inhibit Apoptosis of MC3T3-E1 Cells Induced by High Glucose and Promote Osteogenic Differentiation Through Wnt/β-Catenin Signaling Pathway. Mol. Biotechnol..

[54] Yang N., Zhang N., Lu G., Zeng S., Xing Y., Du L. (2024). RNA-binding proteins potentially regulate the alternative splicing of cell cycle-associated genes in proliferative diabetic retinopathy. Sci. Rep..

[55] Stubbins R.J., Francis A., Kuchenbauer F., Sanford D. (2022). Management of Acute Myeloid Leukemia: A Review for General Practitioners in Oncology. Curr. Oncol..

[56] Guo Z., Zhuang H., Shi X. (2024). Therapeutic efficacy of ferroptosis in the treatment of colorectal cancer (Review). Oncol. Lett..

[57] Fukushima C.M., de Groot J. (2024). Updates for newly diagnosed and recurrent glioblastoma: A review of recent clinical trials. Curr. Opin. Neurol..

[58] Afghani N., Mehta T., Wang J., Tang N., Skalli O., Quick Q.A. (2017). Microtubule actin cross-linking factor 1, a novel target in glioblastoma. Int. J. Oncol..

[59] Cavaliere M., Bisogno A., Scarpa A., D’Urso A., Marra P., Colacurcio V., De Luca P., Ralli M., Cassandro E., Cassandro C. (2021). Biomarkers of laryngeal squamous cell carcinoma: A review. Ann. Diagn. Pathol..

[60] Warth A., Muley T., Meister M., Stenzinger A., Thomas M., Schirmacher P., Schnabel P.A., Budczies J., Hoffmann H., Weichert W. (2012). The novel histologic International Association for the Study of Lung Cancer/American Thoracic Society/European Respiratory Society classification system of lung adenocarcinoma is a stage-independent predictor of survival. J. Clin. Oncol..

[61] May J.M., Bylicky M., Chopra S., Coleman C.N., Aryankalayil M.J. (2021). Long and short non-coding RNA and radiation response: A review. Transl. Res..

[62] Dong H.X., Wang R., Jin X.Y., Zeng J., Pan J. (2018). LncRNA DGCR5 promotes lung adenocarcinoma (LUAD) progression via inhibiting hsa-mir-22-3p. J. Cell Physiol..

[63] Liu Y., Yang C., Cao C., Li Q., Jin X., Shi H. (2020). Hsa_circ_RNA_0011780 Represses the Proliferation and Metastasis of Non-Small Cell Lung Cancer by Decreasing FBXW7 via Targeting miR-544a. OncoTargets Ther..

[64] Natarelli N., Aleman S.J., Mark I.M., Tran J.T., Kwak S., Botto E., Aflatooni S., Diaz M.J., Lipner S.R. (2024). A Review of Current and Pipeline Drugs for Treatment of Melanoma. Pharmaceuticals.

[65] Lumish M.A., Kohn E.C., Tew W.P. (2024). Top advances of the year: Ovarian cancer. Cancer.

[66] Zhang J., Zhang Y., Wang J., Xia Y., Zhang J., Chen L. (2024). Recent advances in Alzheimer’s disease: Mechanisms, clinical trials and new drug development strategies. Signal Transduct. Target. Ther..

[67] Ka M., Jung E.M., Mueller U., Kim W.Y. (2014). MACF1 regulates the migration of pyramidal neurons via microtubule dynamics and GSK-3 signaling. Dev. Biol..

[68] Wu X., Shen Q.T., Oristian D.S., Lu C.P., Zheng Q., Wang H.W., Fuchs E. (2011). Skin stem cells orchestrate directional migration by regulating microtubule-ACF7 connections through GSK3β. Cell.

[69] Kim T., Vidal G.S., Djurisic M., William C.M., Birnbaum M.E., Garcia K.C., Hyman B.T., Shatz C.J. (2013). Human LilrB2 is a β-amyloid receptor and its murine homolog PirB regulates synaptic plasticity in an Alzheimer’s model. Science.

[70] Qian W., Shi J., Yin X., Iqbal K., Grundke-Iqbal I., Gong C.X., Liu F. (2010). PP2A regulates tau phosphorylation directly and also indirectly via activating GSK-3β. J. Alzheimer’s Dis..

[71] Sanabria-Castro A., Alvarado-Echeverria I., Monge-Bonilla C. (2017). Molecular Pathogenesis of Alzheimer’s Disease: An Update. Ann. Neurosci..

[72] Kato T. (2019). Current understanding of bipolar disorder: Toward integration of biological basis and treatment strategies. Psychiatry Clin. Neurosci..

[73] Gordovez F.J.A., McMahon F.J. (2020). The genetics of bipolar disorder. Mol. Psychiatry.

[74] Dobyns W.B., Aldinger K.A., Ishak G.E., Mirzaa G.M., Timms A.E., Grout M.E., Dremmen M.H.G., Schot R., Vandervore L., van Slegtenhorst M.A. (2018). MACF1 Mutations Encoding Highly Conserved Zinc-Binding Residues of the GAR Domain Cause Defects in Neuronal Migration and Axon Guidance. Am. J. Hum. Genet..

[75] Wang Q., Li M., Yang Z., Hu X., Wu H.M., Ni P., Ren H., Deng W., Li M., Ma X. (2015). Increased co-expression of genes harboring the damaging de novo mutations in Chinese schizophrenic patients during prenatal development. Sci. Rep..

[76] Zhang Y., Yin C., Hu L., Chen Z., Zhao F., Li D., Ma J., Ma X., Su P., Qiu W. (2018). MACF1 Overexpression by Transfecting the 21 kbp Large Plasmid PEGFP-C1A-ACF7 Promotes Osteoblast Differentiation and Bone Formation. Hum. Gene Ther..

[77] Zanotti S., Smerdel-Ramoya A., Canalis E. (2011). HES1 (hairy and enhancer of split 1) is a determinant of bone mass. J. Biol. Chem..

[78] Hilton M.J., Tu X., Wu X., Bai S., Zhao H., Kobayashi T., Kronenberg H.M., Teitelbaum S.L., Ross F.P., Kopan R. (2008). Notch signaling maintains bone marrow mesenchymal progenitors by suppressing osteoblast differentiation. Nat. Med..

[79] Padial-Molina M., de Buitrago J.G., Sainz-Urruela R., Abril-Garcia D., Anderson P., O’Valle F., Galindo-Moreno P. (2019). Expression of Musashi-1 During Osteogenic Differentiation of Oral MSC: An In Vitro Study. Int. J. Mol. Sci..

[80] Stojanovic N., Dekanic A., Paradzik M., Majhen D., Ferencak K., Ruscic J., Bardak I., Supina C., Tomicic M.T., Christmann M. (2018). Differential Effects of Integrin α v Knockdown and Cilengitide on Sensitization of Triple-Negative Breast Cancer and Melanoma Cells to Microtubule Poisons. Mol. Pharmacol..

[81] Filadi R., Pendin D., Pizzo P. (2018). Mitofusin 2: From functions to disease. Cell Death Dis..

[82] Hu L., Su P., Li R., Yan K., Chen Z., Shang P., Qian A. (2015). Knockdown of microtubule actin crosslinking factor 1 inhibits cell proliferation in MC3T3-E1 osteoblastic cells. BMB Rep..

[83] Hu L., Su P., Yin C., Zhang Y., Li R., Yan K., Chen Z., Li D., Zhang G., Wang L. (2018). Microtubule actin crosslinking factor 1 promotes osteoblast differentiation by promoting β-catenin/TCF1/Runx2 signaling axis. J. Cell. Physiol..

[84] Yin C., Zhang Y., Hu L., Tian Y., Chen Z., Li D., Zhao F., Su P., Ma X., Zhang G. (2018). Mechanical unloading reduces microtubule actin crosslinking factor 1 expression to inhibit β-catenin signaling and osteoblast proliferation. J. Cell. Physiol..

[85] Zhao F., Ma X., Qiu W., Wang P., Zhang R., Chen Z., Su P., Zhang Y., Li D., Ma J. (2020). Mesenchymal MACF1 Facilitates SMAD7 Nuclear Translocation to Drive Bone Formation. Cells.

[86] Eskildsen T., Taipaleenmaki H., Stenvang J., Abdallah B.M., Ditzel N., Nossent A.Y., Bak M., Kauppinen S., Kassem M. (2011). MicroRNA-138 regulates osteogenic differentiation of human stromal (mesenchymal) stem cells in vivo. Proc. Natl. Acad. Sci. USA.

[87] Shirakawa J., Ezura Y., Moriya S., Kawasaki M., Yamada T., Notomi T., Nakamoto T., Hayata T., Miyawaki A., Omura K. (2014). Migration linked to FUCCI-indicated cell cycle is controlled by PTH and mechanical stress. J. Cell. Physiol..

[88] Han Y., Kim C.Y., Cheong H., Lee K.Y. (2016). Osterix represses adipogenesis by negatively regulating PPARγ transcriptional activity. Sci. Rep..

[89] Zaoui K., Benseddik K., Daou P., Salaun D., Badache A. (2010). ErbB2 receptor controls microtubule capture by recruiting ACF7 to the plasma membrane of migrating cells. Proc. Natl. Acad. Sci. USA.

[90] Liu P., Wakamiya M., Shea M.J., Albrecht U., Behringer R.R., Bradley A. (1999). Requirement for Wnt3 in vertebrate axis formation. Nat. Genet..

[91] Kelly O.G., Pinson K.I., Skarnes W.C. (2004). The Wnt co-receptors Lrp5 and Lrp6 are essential for gastrulation in mice. Development.

[92] Castanon M.J., Walko G., Winter L., Wiche G. (2013). Plectin-intermediate filament partnership in skin, skeletal muscle, and peripheral nerve. Histochem. Cell Biol..

[93] Lin P., Cao W., Chen X., Zhang N., Xing Y., Yang N. (2024). Role of mRNA-binding proteins in retinal neovascularization. Exp. Eye Res..

